# Evaluation of the Cardiotoxicity of Mitragynine and Its Analogues Using Human Induced Pluripotent Stem Cell-Derived Cardiomyocytes

**DOI:** 10.1371/journal.pone.0115648

**Published:** 2014-12-23

**Authors:** Jun Lu, Heming Wei, Jianjun Wu, Mohd Fadzly Amar Jamil, Mei Lan Tan, Mohd Ilham Adenan, Philip Wong, Winston Shim

**Affiliations:** 1 National Heart Research Institute Singapore, National Heart Centre Singapore, Singapore, Republic of Singapore; 2 Cardiovascular & Metabolic Disorders Program, DUKE-NUS Graduate Medical School Singapore, Singapore, Republic of Singapore; 3 Malaysian Institute of Pharmaceuticals & Nutraceuticals. Ministry of Science, Technology & Innovation (MOSTI), Pulau Pinang, Malaysia; 4 Advanced Medical and Dental Institute, Universiti Sains Malaysia, Pulau Pinang, Malaysia; Centro Cardiologico Monzino, Italy

## Abstract

**Introduction:**

Mitragynine is a major bioactive compound of Kratom, which is derived from the leave extracts of Mitragyna speciosa Korth or *Mitragyna speciosa* (*M. speciosa*), a medicinal plant from South East Asia used legally in many countries as stimulant with opioid-like effects for the treatment of chronic pain and opioid-withdrawal symptoms. Fatal incidents with Mitragynine have been associated with cardiac arrest. In this study, we determined the cardiotoxicity of Mitragynine and other chemical constituents isolated using human induced pluripotent stem cell-derived cardiomyocytes (hiPSC-CMs).

**Methods and Results:**

The rapid delayed rectifier potassium current (*I*_Kr_), L-type Ca^2+^ current (*I*_Ca,L_) and action potential duration (APD) were measured by whole cell patch-clamp. The expression of KCNH2 and cytotoxicity was determined by real-time PCR and Caspase activity measurements. After significant *I*_Kr_ suppression by Mitragynine (10 µM) was confirmed in hERG-HEK cells, we systematically examined the effects of Mitragynine and other chemical constituents in hiPSC-CMs. Mitragynine, Paynantheine, Speciogynine and Speciociliatine, dosage-dependently (0.1∼100 µM) suppressed *I*_Kr_ in hiPSC-CMs by 67% ∼84% with IC_50_ ranged from 0.91 to 2.47 µM. Moreover, Mitragynine (10 µM) significantly prolonged APD at 50 and 90% repolarization (APD50 and APD90) (439.0±11.6 vs. 585.2±45.5 ms and 536.0±22.6 vs. 705.9±46.1 ms, respectively) and induced arrhythmia, without altering the L-type Ca^2+^ current. Neither the expression,and intracellular distribution of KCNH2/Kv11.1, nor the Caspase 3 activity were significantly affected by Mitragynine.

**Conclusions:**

Our study indicates that Mitragynine and its analogues may potentiate Torsade de Pointes through inhibition of *I*_Kr_ in human cardiomyocytes.

## Introduction

Kratom is derived from leave extracts of Mitragyna speciosa Korth (*M. speciosa*), which is widely found in tropical and subtropical regions of Asia and it has been used as folk medicine since the 19th century. [1]–[3] It is known to have opiate- and cocaine-like effects and has been traditionally used as a stimulant, analgesia, and a substitute for opiate addicts to alleviate withdrawal symptoms and other conditions. [4]–[8] Other known applications of Kratom include anti-inflammatory, antipyretic, antitussive, antihypertensive, local anesthetic, and hypoglycemic control [2], [9]–[11].

Kratom is legally accessible without a prescription in many countries and remains popular for recreational use in the United Kingdom. [12] Long-term administration of Kratom has been associated with opioid misuse, abuse, dependence and addiction [6] with symptoms such as anorexia, weight loss and insomnia. [13]–[14] Kratom and its related products have been associated with drug toxicity with life-threatening consequences [12] including seizure. [15] Death from drug toxicity has been reported in which Kratom was used together with O-desmethyltramadol [16] and Propylhexedrine. [17] However, cardiotoxicity of Kratom and its derivatives is not well understood.

Drug-induced blockage of the human Ether-à-go-go-Related Gene (hERG) channel in the heart is a major risk of cardiotoxicity. hERG or KCNH2 encodes the alpha subunit of a potassium ion channel that mediates the rapid outward delayed rectifier potassium current (*I*_Kr_). [18] Cardiotoxicity assessment using hERG-overexpressing human embryonic kidney (HEK) cells has been commonly utilized though human cardiomyocytes may be a more pertinent model as both *I*_Kr_ and the action potential duration (APD) could be determined. However, cardiomyocytes from human heart are virtually unavailable due to technical hurdles and safety concerns.

Recent progress in generating human induced pluripotent stem cells (hiPSCs) from somatic cells by reprogramming helps to create a unique source of functional human cardiomyocytes named hiPSC-derived cardiomyocytes (hiPSC-CMs) generated via cardiomyogenic differentiation of hiPSCs. [19] hiPSC-CMs have ionic currents characteristics resembling those reported for adult human cardiomyocytes. [20], [21] They have been successfully adopted in disease modeling of long QT syndrome (LQTS) and drug testing [22].

In this study, we investigate the cardiotoxicty of Mitragynine and its analogues by studying their effects on hERG and APD. Our data show that Mitragynine and its analogues, at concentrations close to the plasma levels reported in lethal cases, [16], [23] exert a significant cardiotoxicity by inhibiting hERG current, prolonging APD and inducing arrhythmia.

## Materials and Methods

### Isolation of Mitragynine and its analogues

**Figure 1:**
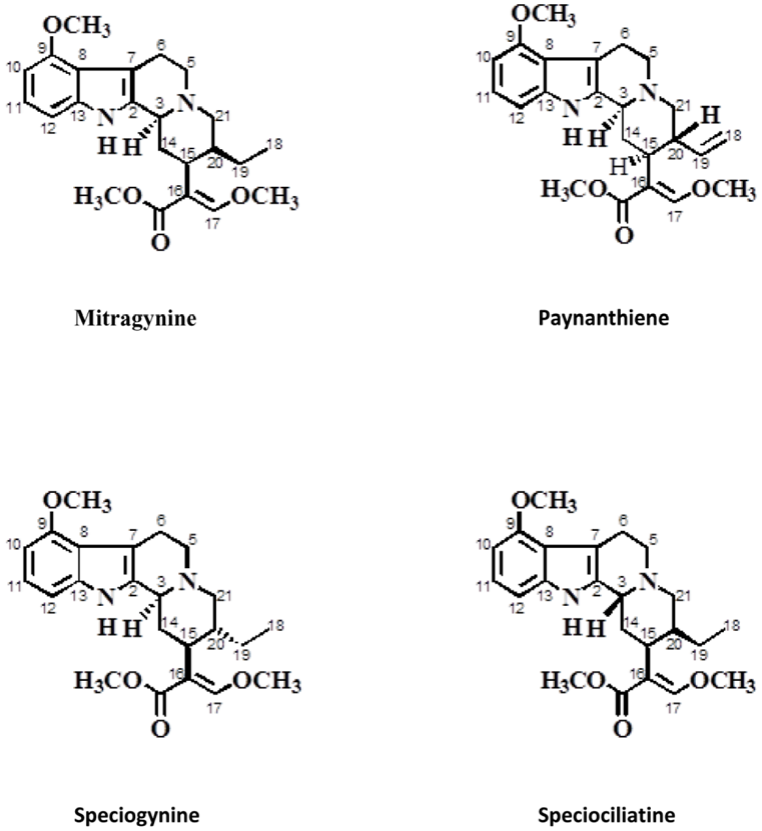
Structure of Mitragynine and analogues.

The fresh leaves of M. speciosa Korth (Rubiaceae) were collected from the state of Perlis, Malaysia, with permission from the Narcotic Unit, Royal Malaysian Police (PDRM). The approval to carry out research on Mitragynine was obtained from the Ministry of Health, Malaysia. Detailed method for isolation and purification of Mitragynine and its analogues was shown in S1 Methods. As previously described by Takayama and co-workers, [24] the naturally occurring indole alkaloid, Mitragynine and its analogues, Paynanthiene, Speciogynine, and Speciociliatine, belong to the class of corynanthe alkaloids. The structure and identity of the compounds were confirmed using 1H**-**NMR and 13C**-**NMR analysis (see S1 Methods). The molecular structures of these compounds are shown in Fig. 1. In this study, Mitragynine and its analogues were used for cardiotoxicity testing.

### Cells and cell culture

Stable transgenic hERG-HEK293 cells (hERG-HEK, EZCells^TM^) were purchased from ChanTest (Cleveland, USA) and maintained following the manufacturer’s instruction. Human hiPSC-CMs (iCell Cardiomyocytes and hereby named iCell) were obtained from Cellular Dynamics International Inc (Madison, USA). Detail cultural methods were described in the S1 Methods.

### Patch-clamp assays

Patch-clamp technique was performed for recording K^+^ and Ca^2+^ currents and action potentials. The signals were amplified using Axon 200B patch clamp amplifier (Axon instrument, USA) and low-pass filtered at 5 kHz. Data acquisition was achieved using the Digidata 1440A (Axon instruments Inc, USA). Borosilicate glass electrodes (1.5 mm OD) were pulled by a horizontal puller (Model P-97, Sutter Instrument, USA) and fired-polished to a final resistance of 2**–**3 MΩ when filled with internal solution. After a gigaohm-seal was obtained by negative pressure suction, the cell membrane was ruptured by a gentle suction to establish whole-cell configuration with seal resistance >1000 MΩ. The series resistance was compensated by 50**–**70**%** to minimize voltage errors. Cardiomyocyte cell membrane capacitance averaged 64.3±2.5 pF. Cells were maintained at 35**–**37°C during the recording by a temperature controller (Warner Instruments) except for *I*_Ca,L_ recordings that were performed at room temperature.

For current clamp experiments, APs were recorded in hiPSC-CMs under current gate in normal Tyrode’s solution containing (in mM): NaCl 140, KCl 5.4, CaCl_2_ 1.8, MgCl_2_ 1, glucose 10, HEPES10, adjusted to pH 7.40 with NaOH. Pipettes solution contained (in mM): KCl 130, NaCl 5, MgCl_2_ 1, MgATP 3, EGTA 10, and HEPES 10, adjusted to pH 7.20 with KOH. hiPSC-CMs were paced at 0.2 and 1.0 Hz.

For voltage clamp experiments of hiPSC-CMs, the external solution used for measurements *I*_Kr_ was composed of the following (in mM): NaCl 140, KCl 5.4, CaCl_2_ 1.8, MgCl_2_ 1, glucose 10, HEPES 10, Nifedipine 0.001, Chromanol 293B 0.01, adjusted to pH 7.40 with NaOH. [25], [26] For recording *I*_Ca,L_, external solution contained (in mM): NaCl 140, CsCl 10, CaCl_2_ 1.8, MgCl_2_ 1, glucose 10, HEPES 10, adjusted to pH 7.40 with NaOH. Pipettes solution for *I*_Kr_ recordings contained (in mM): KCl 130, MgCl_2_ 1, MgATP 3, EGTA 10, and HEPES 10, adjusted to pH 7.20 with KOH. Pipettes solution for *I*_Ca,L_ recordings contained (in mM): CsCl 120, MgCl_2_ 3, MgATP 5, EGTA 10, HEPES 5, adjusted to pH 7.20 with CsOH. Ion currents were analyzed and fitted using the Clampfit 10.0 (Axon Instruments Inc, USA) and Origin 7.0 software (Originlab Corporation).

To measure the activation time constant of voltage-activated K^+^ current, *I*_kr_ in hiPSC-CMs (n = 4) was elicited by a step to 20 mV for 2 s from a holding potential of–50 mV, and the tail current was recorded after the step to −50 mV lasting 4 s. To measure the activation time constant of voltage-activated L type Ca^2+^ current, *I*_Ca,L_ in hiPSC-CMs (n = 4) was clamped to 10 mV from a holding potential of −70 mV for 500 ms.

Polymerase Chain Reaction (RT-PCR), Caspase 3 activity measurement and immunocytochemistry.

Detail methods were described in **S1** **Methods** and **S1 Table**.

### Statistical analysis

Data are expressed as mean ± SEM. Statistical significance was analyzed using a Student *t* test or one-way ANOVA followed up with Tukey tests. *P*<0.05 was considered statistically significant.

## Results

### Mitragynine suppressed *I*_kr_ in hERG-expressing HEK293 cells

**Figure 2:**
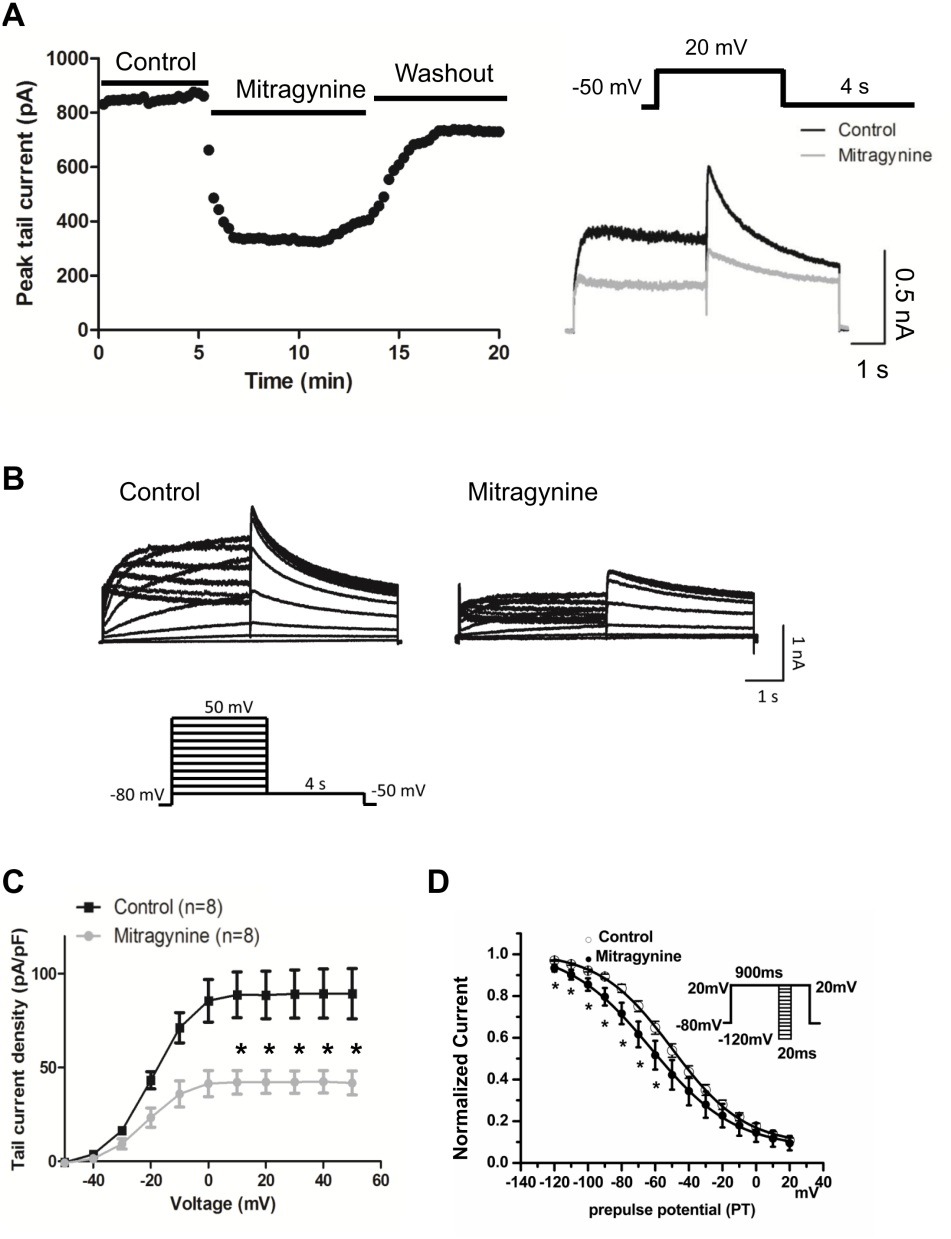
Mitragynine suppressed *I*_Kr_ in hERG-HEK cells. The effect of Mitragynine was tested in hERG-HEK cells. (**A**) The time-course and wash out of Mitragynine (n = 4). The insert is the voltage protocol used to elicit hERG. (**B**) A representative *I*_kr_ peak tail current traces recorded with Mitragynine (10 µM) treatment. The insert is the voltage protocol used to elicit hERG. (**C**) The I-V curve of *I*_kr_ tail currents recorded in hERG-HEK cells prior to and post Mitragynine treatment. *p<0.05. Data represent are the whole cell currents (raw current) uncorrected for the presence of background currents. (**D**) The steady-state inactivation properties of *I*_Kr_ in hERG-HEK293 cells. Conditioning pulses between −120 and +20 mV in 10 mV increments for 20 milliseconds were applied after a depolarizing pulse to +20 mV for 900 milliseconds, followed by a common test pulse to +20 mV. The voltage protocol is illustrated in the inset. *p<0.05 (n = 5). The voltage protocol is illustrated in the inset.

The effect of Mitragynine was tested in hERG-HEK cells (**Fig. 2**). Compared with control, Mitragynine (10 µM) treated cells demonstrated a significant reduction in tail current amplitude (pA/pF) measured at 10, 20, 30, 40, and 50 mV (88.66±12.21 vs. 42.08±6.32; 88.63±12.61 vs. 42.25±6.09; 89.17±12.84 vs. 42.24±5.95; 89.36±13.10 vs. 42.36±6.04; and 89.32±13.38 vs. 41.75±6.40; n = 8, *P*<0.05) (**Fig. 2C**). Moreover, it was noted that Mitragynine (10 µM) treatment significantly shifted the voltage-dependent steady-state inactivation curve towards more negative potentials with a ∼13 mV shift in half-inactivation voltage documented (V_1/2_ −49.5±2.8 mV in controls and −62.3±5.0 mV after Mitragynine, *P*<0.05, n = 8). Slope factors did not differ significantly (**Fig. 2D**). For measuring the steady-state inactivation, conditioning pulses between −120 and +20 mV in 10 mV increments for 20 milliseconds were applied after a depolarizing pulse to +20 mV for 900 milliseconds, followed by a common test pulse to +20 mV. The voltage protocol is illustrated in the inset. More protocols for the voltage clamp of hERG-HEK293 and data analysis were described in the **S1 Methods**.

### Mitragynine and its analogues suppressed *I*_kr_ in hiPSC-CMs

In human cardiomyocytes, *I*_Kr_ is a major component in the tail current of the outward rectifier potassium currents (*I*_K_). *I*_Kr_ currents were elicited by depolarizing steps between −40 mV and 50 mV with a 10 mV increments for 2 s from the holding potential at −50 mV. Tail current was recorded after the test potential was reverted to −50 mV lasting for 4 s. The *I*_Kr_ currents activation curves were generated by plotting the normalized tail current amplitudes against the step potentials and were fitted with a Boltzmann equation: *I* = *I_max_*×[1+exp(*V_1/2_*–*V*)/*κ*]^−1^, where I*_max_* is maximum amplitude, *V_1/2_* and *κ* are half-activation voltage and the slope factor. *I*_Kr_ was activated at −40 mV and reaching maximum at approximately 0 mV and at more positive voltages inward rectification was present because of voltage-dependent rapid inactivation.

To determining the steady-state inactivation properties of the *I*_kr_ (hERG) in hiPSC-CMs, conditioning pulses between −130 and +30 mV in 10 mV increments for 5 milliseconds were applied after a depolarizing pulse to +30 mV for 900 milliseconds, followed by a common test pulse to +30 mV. The corrected steady-state inactivation curves were fitted with a Boltzmann function in the following form: I/(I_max_–I_min_) = I/{1+exp[(V_t_–V_1/2_)/K]}+I_min_. Where I is the amplitude of inactivating current corrected for deactivation, I_max_ is the maximum of I, I_min_ is the minimum of I, V_t_ is the pre-pulse of test potential, V_1/2_ is the voltage at which I is half of max, and k is the slope factor. Experiments were performed at room temperature.

**Figure 3:**
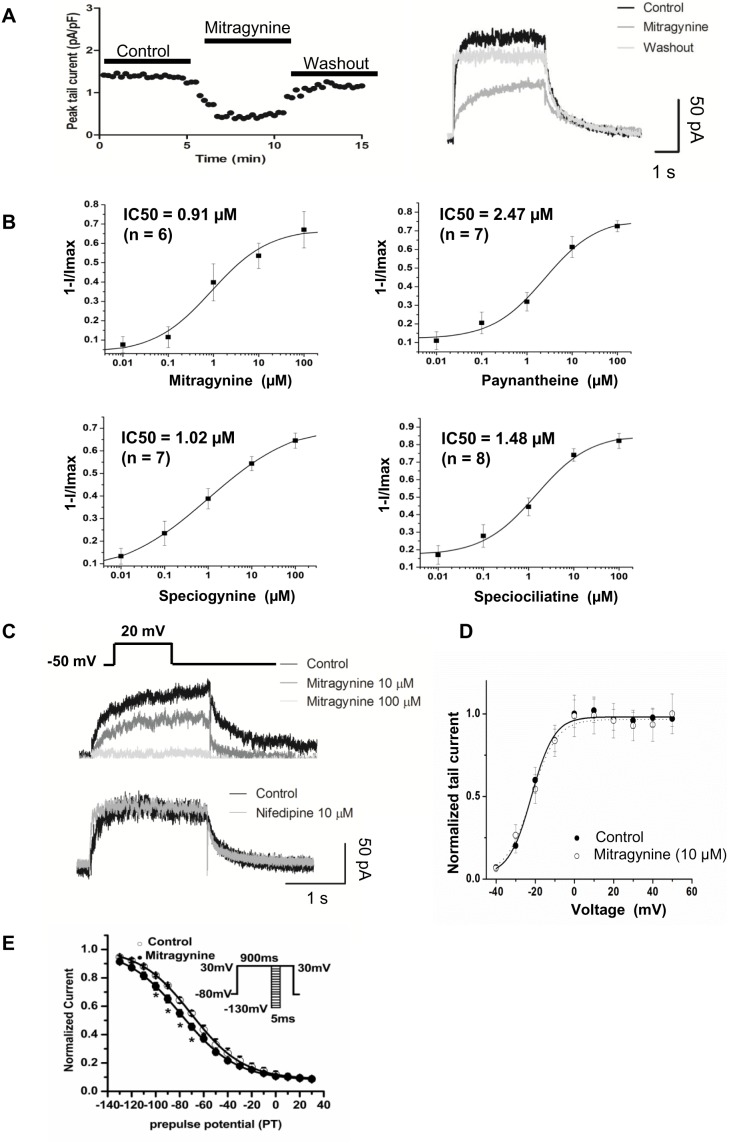
Mitragynine and its analogues suppressed *I*_Kr_ in hiPSC-CMs. (**A**) The time-course and wash out experiments in Mitragynine (n = 4). (**B**) The concentration dependence relationships of *I*_kr_ peak tail current to Mitragynine, Paynantheine, Speciogynine and Speciociliatine with IC50 registered as 0.91, 2.47, 1.02 and 1.48 µM, respectively. (**C**) Voltage protocol (upper) used to elicit hERG and *I*_k_ peak tail current traces with Mitragynine (middle) and nifedipine (bottom) exposure. Representative traces represent the whole cell currents (raw current) uncorrected for the presence of background currents. (**D**) Normalized steady-state activation curves of *I*_Kr_ tail current prior to and post application of 10 µM Mitragynine (n = 5). (E) The steady-state inactivation properties of *I*_Kr_ in hiPSC-CMs. Conditioning pulses between −130 and +30 mV in 10 mV increments for 5 milliseconds were applied after a depolarizing pulse to +30 mV for 900 milliseconds, followed by a common test pulse to +30 mV. The voltage protocol is illustrated in the inset. *p<0.05 (n = 5).

The effects of Mitragynine and its analogues on *I*_Kr_ were measured in hiPSC-CMs (**Fig. 3**) hERG currents were activated with a step to 20 mV for 2 s, and tail current was recorded after stepping to −50 mV for 4 s. *I*_Kr_ was quantified by fitting the Hill equation: *I*_compound_/*I*_control_ = 1/[1+(D/IC50)^n^], where D is the compound concentration, IC_50_ is the drug concentration for 50% inhibition, and n is the Hill coefficient. Cultured hiPSC-CMs were exposed to Mitragynine, Paynantheine, Speciogynine and Speciociliatine at 0.01, 0.1, 1, 10 and 100 µM and *I*_kr_ was recorded 2 minutes later. The IC_50_ for all 4 compounds were 0.91, 2.47, 1.02 and 1.48 µM, respectively (**Fig. 3B**). At 100 µM, Mitragynine, Paynantheine, Speciogynine and Speciociliatine registered a maximum inhibition of the *I*_K_ tail current by 67.0±9.41%, 69.91±3.09%, 68.01±3.95%, and 83.74±5.74%, respectively (**Fig. 3B** **,** **Table 1** **)**. All compounds exert similar magnitudes of inhibition on *I*_kr_ tail current. As a negative control, Nifedipine (a L-type Ca^2+^ channel blocker), which is known not to inhibit *I*_kr_, consistently failed to show any significant effects on hERG (**Fig. 3C**). Furthermore, Mitragynine did not shift the half-activation voltage of *I*_kr_ in hiPSC-CMs (**Fig. 3D**). However, similar to that observed in hERG-HEK293 cells, Mitragynine (10 µM) treatment led to a ∼10 mV negative shift in the voltage-dependent steady state inactivation (V_1/2_ −69.15±2.21 mV in controls vs. −79.04±2.64 mV after Mitragynine; *P*<0.05, n = 5) of *I*_kr_. However, slope factor did not differ significantly post treatment (**Fig. 3E**).

**Table 1:** Inhibition of Mitragynine and its analogues on *I*_Kr_ tail current in hiPSC-CMs.

Compounds	% inhibition	0.01 uM	0.1 uM	1 uM	10 uM	100 uM
**Mitragynine**	Ave	7.63%	11.48%	39.83%	53.55%	67.06%
**(n = 12)**	SEM	4.18%	5.37%	9.55%	6.51%	9.41%
	*P* value	0.0716	0.0388	0.0004	0.0000	0.0000
**Paynantheine**	Ave	12.21%	25.71%	32.70%	59.34%	69.91%
**(n = 11)**	SEM	6.79%	7.34%	6.27%	6.77%	3.09%
	*P* value	0.0756	0.0015	0.0000	0.0000	0.0000
**Speciogynine**	Ave	9.81%	21.73%	41.22%	57.24%	68.01%
**(n = 13)**	SEM	3.99%	6.30%	6.01%	3.30%	3.95%
	*P* value	0.0140	0.0032	0.0000	0.0000	0.0000
**Speciociliaitine**	Ave	24.52%	33.77%	42.51%	79.21%	83.74%
**(n = 14)**	SEM	8.28%	9.70%	5.67%	3.53%	5.74%
	*P* value	0.0046	0.0025	0.0000	0.0000	0.0000

The current-voltage relationship for *I*_Kr_ measured at the end of the depolarizing step was determined (**S1B Fig.**). The peak step current density of *I*_Kr_ was 0.83±0.18 pA/pF (**S1B Fig.**, left). The *I*_Kr_ tail current was maximum after voltage steps positive to 0 mV (**S1B Fig.**, right). The half-maximal activation voltage (*V_1/2_*) for *I*_Kr_ was −18.6±0.91 mV. The normalized tail current density/voltage relationships of *I*_Kr_ were in agreement with previously reported characteristic kinetics of *I*_Kr_ [25], [26].

### Mitragynine prolonged action potential duration in hiPSC-CMs

**Figure 4:**
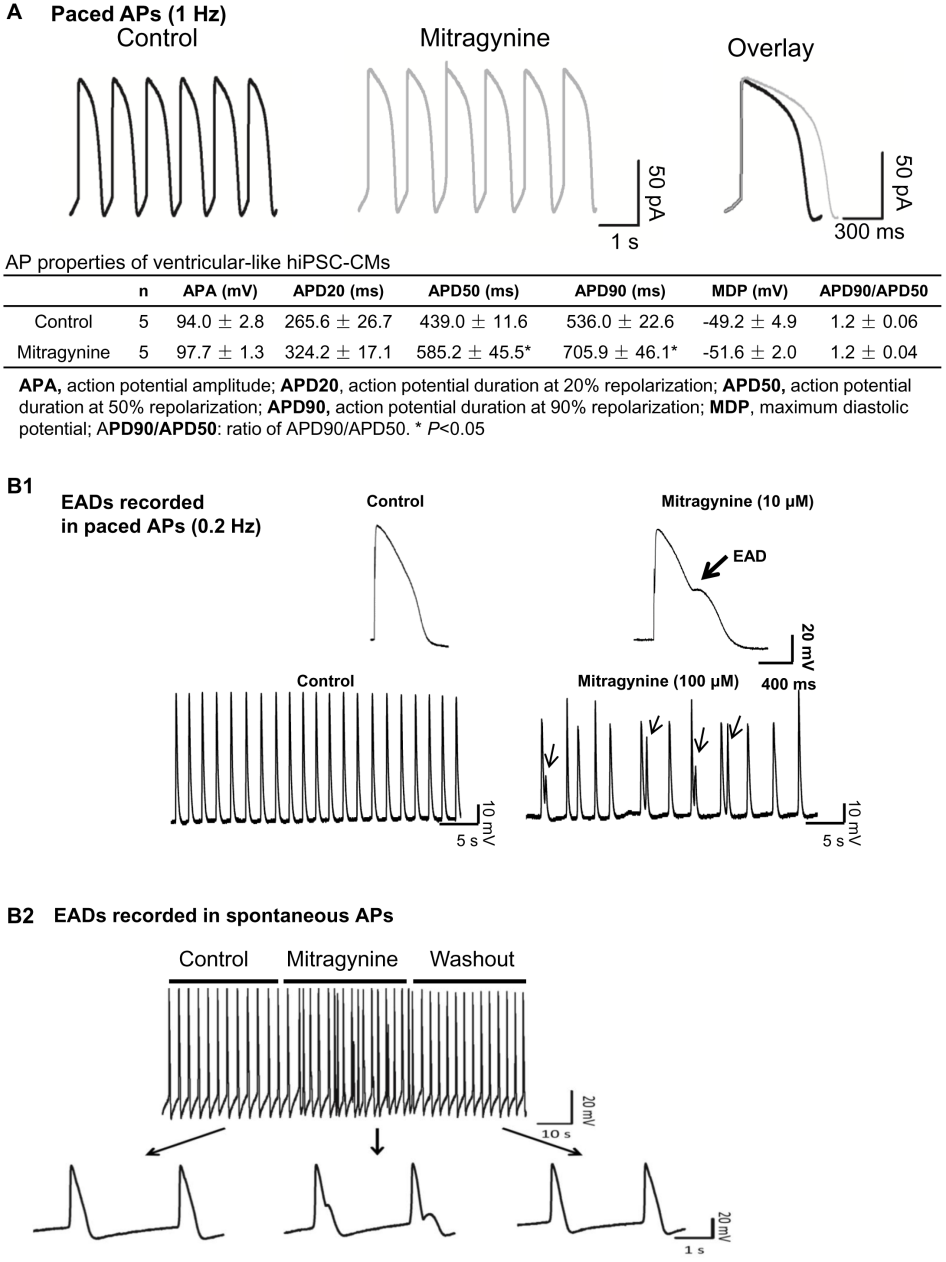
Mitragynine prolonged action potential duration in hiPSC-CMs. (**A**) APs were recorded prior to and post application of 10 µM Mitragynine in ventricular-like hiPSC-CMs paced at 1 Hz. Overlay of single AP from the same cell prior to and post Mitragynine treatment (right) is presented. The table inserted below represents the AP properties. (**B1**) Mitragynine (10 µM) treatment resulted in sporadic early after depolarization (EAD, indicated by arrow) in hiPSC-CM paced at 0.2 Hz. (**B2**) Mitragynine (100 µM) treatment resulted in frequent EADs in spontaneously contracting hiPSC-CMs.

Ventricular-like hiPSC-CMs were identified by their characteristic AP traces (**Fig. 4A** and **S1 Results**). **Fig. 4A** shows the typical AP trace of ventricular-like hiPSC-CMs paced at 1 Hz. Mitragynine (10 µM) significantly prolonged AP duration at 50% and 90% repolarization (APD50 and APD90 respectively). Moreover, sporadic early afterdepolarizations (EADs) was induced by 10 µM Mitragynine in hiPSC-CMs paced at 0.2 Hz (**Fig. 4B** **.1**) and frequent EADs were induced by 100 µM Mitragynine (a concentration that exerted the highest inhibition of hERG) in spontaneously contracting hiPSC-CMs and the EADs disappeared after wash out of Mitragynine (**Fig. 4B** **.2**).

### Mitragynine did not affect the L-type calcium current in hiPSC-CMs

**Figure 5:**
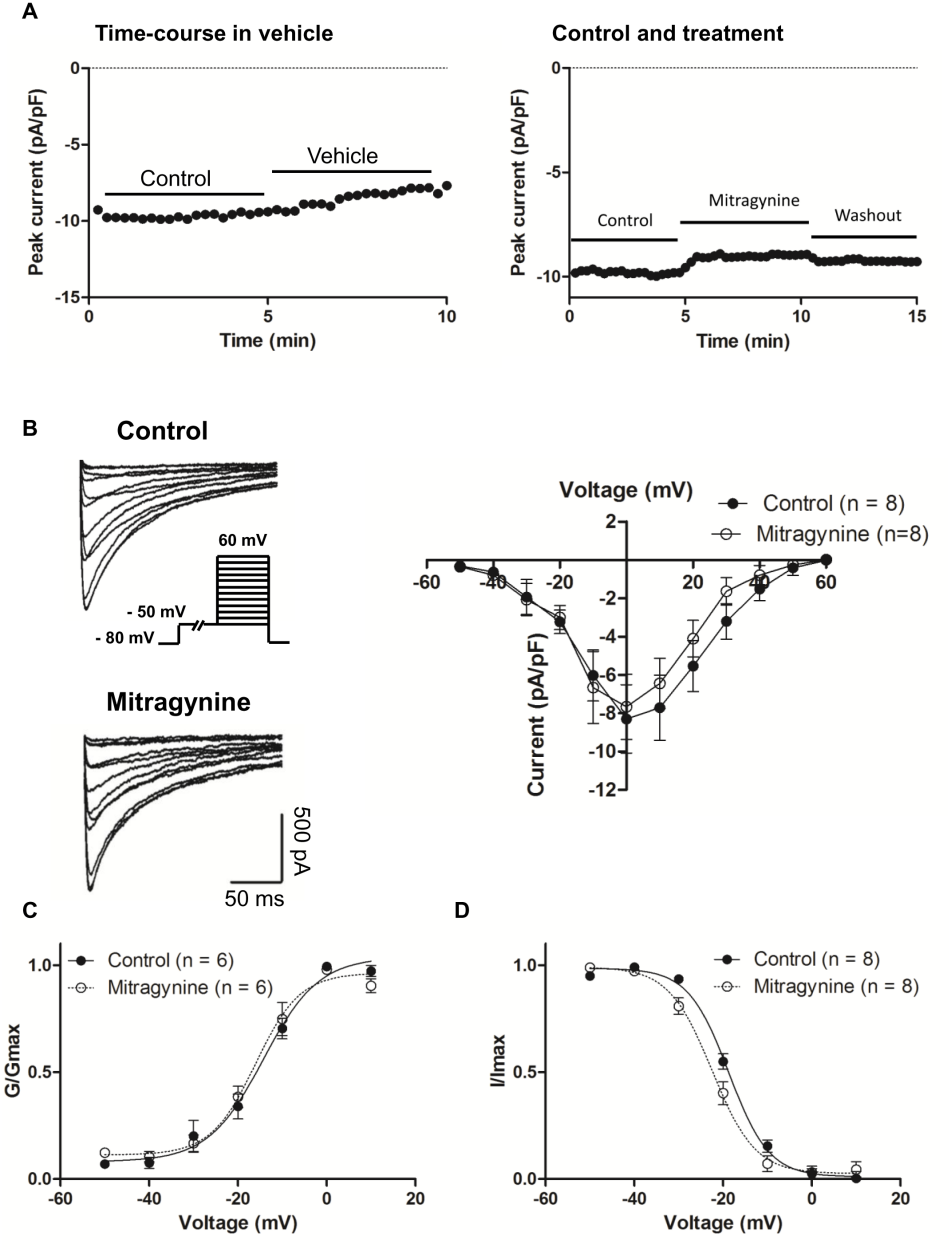
Mitragynine did not alter the L-type calcium current in hiPSC-CMs. (**A**) Time-course in vehicle (left) and wash out of Mitragynine (right), n = 4. (**B**) Left: Representative *I*_Ca,L_ traces prior to and post application of Mitragynine (10 µM). Pulse protocol is shown in the inset. Right: Averaged I-V relations for *I*_Ca,L_ prior to and post Mitragynine. (**C** and **D**) Steady-state activation and inactivation curves of *I*_Ca,L_ prior to and post Mitragynine treatment.

The voltage-gated calcium current, *I*_Ca,L_ is another important contributor to the APD as it contributes to the Phase 2 plateau of the cardiac AP, particularly in ventricular cardiomyocytes. *I*_Ca,L_ activation has also been associated with APD prolongation and a fatal ventricular tachyarrhythmia called Torsade de Pointes (TdP). To exclude the potential effect of Mitragynine on *I*_Ca,L_ that could alter APD, *I*_Ca,L_ was recorded in hiPSC-CMs treated with Mitragynine. Cardiomyocytes were held at −80 mV followed by a 3 s long prepulse at −50 mV to inactivate Na^+^ and T-type Ca^2+^ channels and the recording was performed at room temperature to minimize current rundown. *I*_Ca,L_ was activated by a family of 500 ms depolariztions from −50 mV to +60 mV in 10 mV increments. **Fig. 5A** shows the time-course and wash out of Mitragynine. **Fig. 5B** shows the *I*_Ca,L_ before and after application of Mitragynine. *I*_Ca,L_ was activated at–50 mV and reached a peak at approximately 0 mV. The average current-voltage curve indicated that Mitragynine (10 µM) did not statistically alter the density of *I*_Ca,L_ in hiPSC-CMs (control vs. Mitragynine-treated: −8.49±2.42 pA/pF and −7.67±1.77 pA/pF, n = 6). Next, the voltage-dependent activation was evaluated by plotting normalized Ca^2+^ peak current. Data were fitted with Boltzmann equation. As illustrated in **Fig. 5C**, under control condition, *V_1/2_* and *κ* are −14.5±1.49 mV and 6.13±1.35, which were not significantly different from that of Mitragynine-treated, *V_1/2_* =  −16.2±1.23 mV and *κ  = *4.90±1.03. To measure steady-state inactivation of *I*_Ca, L_, cardiomyocytes were held between −50 mV and +10 mV for 500 ms in 10 mV increments followed by 200 ms long pulse to 10 mV. The current amplitude was normalized to the peak *I*_Ca,L_ and fitted with Boltzmann equation. In **Fig. 5D** **,** *V* *_1/2_* was significantly shifted in the negative direction from −18.3±0.75 mV in controls to–21.4±0.20 mV (*P*<0.01) after Mitragynine treatment. The *κ* are 4.83±0.71 and 5.75±0.19 (*P*<0.05), respectively.

### Mitragynine did not alter the expression of KCNH2/Kv11.1 in hiPSC-CMs and it did not induce apoptosis and cell death

The rapid suppression of hERG by Mitragynine **(**two minutes post drug application) observed in this study indicated that it was unlikely that Mitragynine achieved its effects via altering the synthesis and intracellular trafficking of Kv11.1. In addition, we carried out experiments to exclude potential subacute effect on Kv11.1 expression in hiPSC-CMs at 4 hours exposure to Mitragynine. A concentration of 10 µM of Mitragynine was adopted, which is higher than its IC_50_ *in* *vitro* and the maximal physiological concentration of (1.07 µM, in rats) so to allow Mitragynine to exert more remarkable effects.

**Figure 6:**
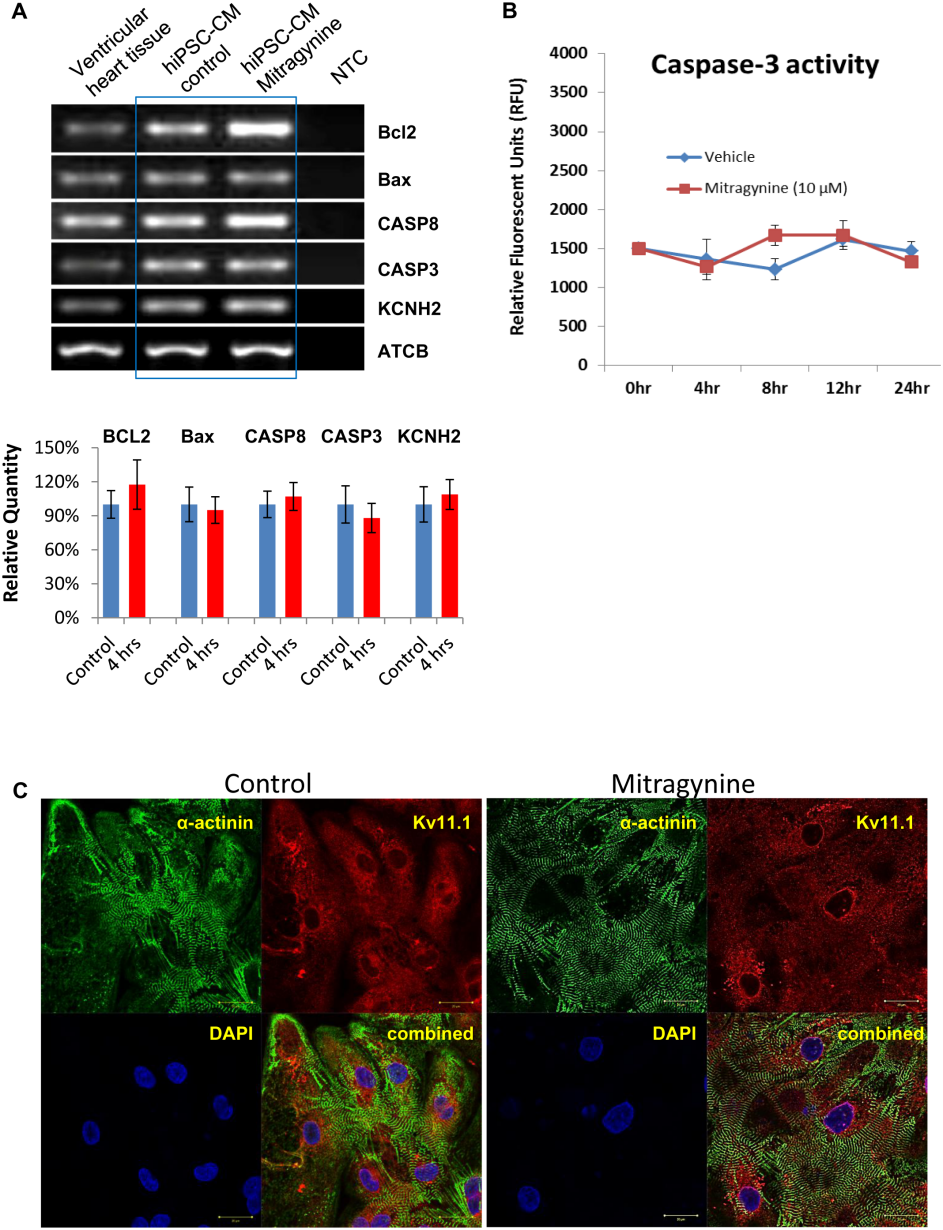
Mitragynine did not alter the expression of KCNH2 and Kv11.1 and the apoptosis-related genes in hiPSC-CMs. (**A**) RT-PCR and real-time qPCR was performed with cDNA prepared from hiPSC-CMs prior to and 4 hours post Mitragynine (10 µM) treatment. (Top) Representative image of PCR products of KCNH2, Caspase 3, Caspase 8, Bcl2, Bax and ACTB are presented Human ventricular heart tissue cDNA was used as control. (**Bottom**) Relative quantity of PCR products of KCNH2, Caspase 3, Caspase 8, Bcl2, Bax measured by qPCR. (**B**) Caspase-3 activity measured in hiPSC-CMs treated with Mitragynine and vehicle (ethanol) for different time periods. (**C**) Representative images (with 3 repeats) of hiPSC-CMs immunofluorescently stained with α-actinin (green) and Kv11.1 (red) are presented. Kv11.1 staining shows similar intensity and distribution of hERG in hiPSC-CMs prior to and 4 hours post Mitragynine (10 µM) treatment. The identity of cardiomyocytes is confirmed by α-actinin staining.

As shown in **Fig. 6A** **,** the expression of KCNH2 was confirmed with human ventricular tissue as well as hiPSC-CMs. The gene expression of KCNH2 in hiPSC-CMs was not altered after Mitragynine treatment (10 µM for 4 hours). Moreover**,** it appeared that Mitragynine treatment neither altered the fluorescence intensity nor the intracellular localization of Kv11.1 in hiPSC-CMs **(** **Fig. 6C**).

Next, the potential cytotoxic effects of Mitragynine on hiPSC-CMs were assessed by measuring the expression levels of apoptotic-related genes. As shown in **Fig. 6A** **Top**, the expressions of Caspase 3, Caspase 8, Bcl2 and Bax genes were confirmed with human ventricular tissue as well as with hiPSC-CMs by RT-PCR. Moreover, qPCR results showed that Mitragynine (10 µM, 4 hours) did not significantly alter the gene expression levels of Caspase 3, Caspase 8, Bcl2 and Bax (**Fig. 6A** **Bottom**). The viability of the cells treated with Mitragynine for 24 hours was examined by Trypan Blue exclusion assay and over 85% viability was observed. In addition, Caspase 3 activity measured in hiPSC-CMs treated with Mitragynine for different time periods demonstrated that there was no significant cell death after treatment (**Fig. 6B**).

## Discussion

Multiple poisoning and fatal cases involving Kratom and Mitragynine have been reported. Unfortunately the underlying causes of these adverse reactions remain unclear. Cases of severe toxicity characterized by seizure were reported on Kratom use together with modafinil and *Datura stramonium.* [6], [15] Drug toxicity death involving Mitragynine has been reported in Sweden in 9 cases over a 1-year time period. [16] The presence of both Mitragynine and O-desmethyltramadol (the active metabolite of tramadol) with the former between 0.05∼0.45 µ (were confirmed in postmortem blood samples. Another fatal case was also attributed to probable Kratom toxicity [23] with presence of benzodiazepines and blood level of Mitragynine at 0.60 mg/L (or 1.51 µa).

Our study provides first scientific evidence of cardiotoxicity of Mitragynine. The IC_50_ of Mitragynine (0.91 µM) observed in our study was close to the drug concentrations measured postmortem though higher doses of Mitragynine were likely to be present in the victims’ system. However, direct evidence of fatal cardiotoxicity of Mitragynine is still lacking. This is compounded by unavailable reference ranges of Mitragynine in plasma as Kratom alkaloids or their metabolites are usually not target analytes of toxicology screening [27].

In the current study, we demonstrate that Kratom/Mitragynine exerts a cardiotoxic effect by inhibiting *I*_Kr_ in hERG-HEK293 and hiPSC-CMs and increasing the APD in the latter with potential of causing TdP. Mitragynine and its analogues at low concentrations (IC_50_ ranging from 0.91 to 2.47µM) potently inhibited *I*_Kr_ in hiPSC-CMs. The unique gating properties of hERG potassium (K^+^) channels play a critical role in cardiac action potential repolarization characterized by relatively slow activation and an unusually fast and voltage-dependent inactivation. Inhibition of hERG has been associated with preferential binding of drugs/reagents to open and/or inactivated states of hERG channels. [28] Our results show that Mitragynine increased the rate of inactivation and caused a marked hyperpolarizing shift in the V1/2 of steady-state inactivation. Such change could lead to reduced *I*_Kr_ and prolonged APD. [29], [30] Mitragynine did not alter the activation of hERG current, suggesting that Mitragynine has a preferential binding to the inactivated state and this is the likely underlying cellular mechanism for potential QT prolongation and TdP in the toxicity of Kratom.

*I*_Ca,L_ is another major contributor of cardiac APD. We found that the effect of Mitragynine on *I*_Ca,L_ in hiPSC-CMs is insignificant although a tendency of *I*_Ca,L_ suppression by Mitragynine was noted.

Reduced hERG channel protein synthesis or trafficking defect represents the alternative yet more chronic cellular mechanism of hERG inhibition and drug-induced torsadogenesis. [31], [32] Such trafficking defects would take hours to develop and thus it requires a long exposure to the drug. In this study, the immediacy of hERG inhibitory effects of Mitragynine and its analogues and the lack of alterations in the gene expression of KCNH2 and intracellular localization of Kv11.1 suggest that the tested compounds did not cause synthesis or trafficking defects of hERG. This is consistent with the reported effects of Kratom and Mitragynine beginning at 5 to 10 min after consumption and lasting for 1 h. [14] In rat models, the time to peak plasma concentration and elimination t½ of Mitragynine are 1.26 and 3.85 h, respectively [33].

At low doses, Kratom produces a stimulant effect. While at higher doses, it exhibits opioid-like effect. [12] Our data indicate that the inhibitory effect of Mitragynine on *I*_Kr_ in hiPSC-CMs was independent of cytotoxic effect as there was no apparent apoptotic effect observed with treated hiPSC-CMs. Our data show that Mitragynine and its analogues exert cardiotoxicity at low concentrations as the IC_50_ values of the compounds for suppressing *I*_Kr_ were far below their half-killing doses observed with many cells lines. Mitragynine has demonstrated half-killing doses ranging from 10.43 ∼1412.06 µM in various cell lines. For example, in neuroblastoma SK-N-SH cell line, the IC_50_ values was 78.90 µM. [34] In RAW 264.7 cells, it was 52.80 µM. [35] Other unpublished IC_50_ values include the Ishikawa cell line (10.40 µM); WRL68 cell line (103.10 µM); HepG2 cell line (20.60 µM); MCF cell line (1412.10 µM).

Cardiotoxicity is one of the main reasons for drug withdrawals and it accounted for 45% of all drugs taken off the market between 1994 and 2006. [36] Cardiotoxicity is commonly characterized by prolongation of the QT interval. Many drugs that prolong QT interval block *I*_kr_, which is responsible for depolarization and contributes to the Phase 3 of the cardiac AP. Thus, current cardiotoxicity testing focuses on hERG/Kv11.1, the ion channel underlying *I*_Kr._ Suppression of hERG/Kv11.1 (*I*_Kr_) can prolong the APD and cause drug-induced LQT syndrome and fatal ventricular tachyarrhythmia or TdP and consequently sudden cardiac death. [37], [38] However, hERG is not the only ion channel that contributes to cardiac APD and accumulating evidences have shown that not all *I*_Kr_ blockers cause QT prolongation or TdP in cardiomyocytes. [39] Therefore, the predictive power of the hERG assay for the torsadogenicity of a specific compound using hiPSC-CMs has great advantage over conventional non-cardiomyocyte cell model such as HEK293.

While the unique value of hiPSC-CMs as in vitro drug testing model has been increasing recognized, [22] some drawbacks should be considered. First, hiPSC-CMs contain different subtypes of cardiomyocytes (mixed with V-, A-, and N-like subtypes). Second, hiPSC-CMs are immature and embryonic-like as compared to adult cardiomyocytes. Nevertheless, as the majority of hiPSC-CMs are ventricular type (∼70%), which is ideally suited for cardiotoxicity testing, their usefulness has been demonstrated in high throughput screening using automatic patch clamp technique [40].

In summary, our study using transgenic hERG-HEK293 and human cardiomyocytes indicate that Mitragynine and its analogues may induce potentially fatal TdP by suppressing hERG-mediated K^+^ currents and prolonging APD. Our study confirms the usefulness of human iPSC-CMs as a model in a comprehensive evaluation of cardiotoxicity of natural compounds.

## Supporting Information

S1 Fig **The I-V relations for** ***I*** **_Kr_ in hiPSC-CMs. (A)** The time-course experiment in vehicle (n = 4). (**B**) Averaged I-V relations for *I*_Kr_ at the end of the depolarizing step. Left and middle, step and tail *I*_Kr_ normalized to maximal current following repolarization to –50 mV.
(TIF)

S1 Table **PCR Primers.** hCASP3, human caspase 3. hCASP8, human caspase 8. hBcl2, human B-cell CLL/lymphoma 2. hBax, human BCL2-associated X protein. ACTB: β-actin.
(DOCX)

S1 Methods (DOCX)

S1 Results (DOCX)
