# Supplementary figures and images for: Evaluation of the Cardiotoxicity of Mitragynine and Its Analogues Using Human Induced Pluripotent Stem Cell-Derived Cardiomyocytes
Source: PLoS One. 2014 Dec 23;9(12):e115648. doi: 10.1371/journal.pone.0115648 (PMC4275233; doi:10.1371/journal.pone.0115648)

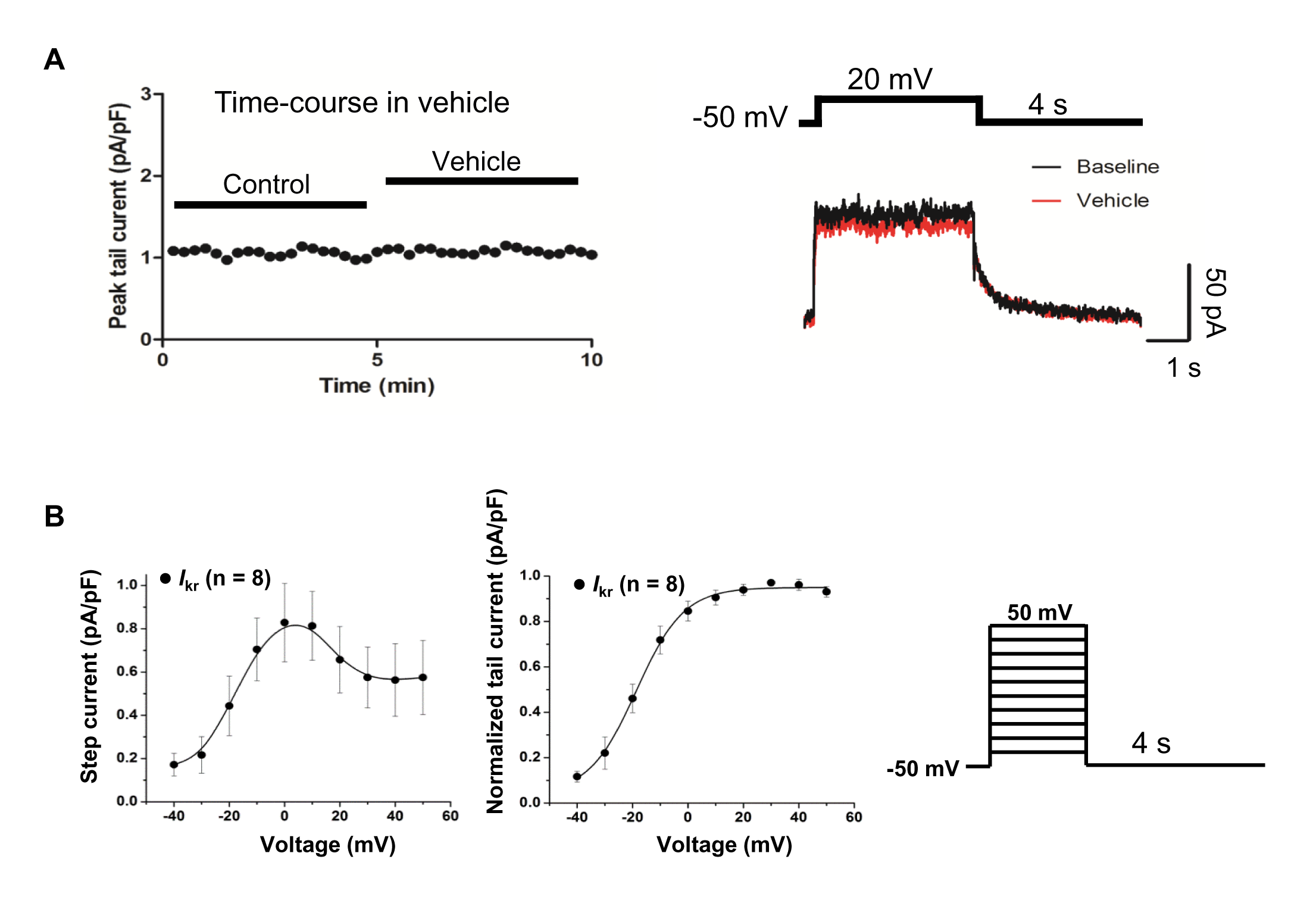

Supplement: S1 Fig — The I-V relations for I Kr in hiPSC-CMs. (A) The time-course experiment in vehicle (n = 4). (B) Averaged I-V relations for I Kr at the end of the depolarizing step. Left and middle, step and tail I Kr normalized to maximal current following repolarization to –50 mV. (TIF) [file pone.0115648.s001.tif]
