# Supplementary material for: Evaluation of the Cardiotoxicity of Mitragynine and Its Analogues Using Human Induced Pluripotent Stem Cell-Derived Cardiomyocytes
Source: PLoS One. 2014 Dec 23;9(12):e115648. doi: 10.1371/journal.pone.0115648 (PMC4275233; doi:10.1371/journal.pone.0115648)
Supplement: S1 Table — PCR Primers. hCASP3, human caspase 3. hCASP8, human caspase 8. hBcl2, human B-cell CLL/lymphoma 2. hBax, human BCL2-associated X protein. ACTB: β-actin. (DOCX) [file pone.0115648.s002.docx]

| **GenBank accession No.** | **Gene** | **Forward primer** | **Reverse primer** | **Product size(bp)** |
| --- | --- | --- | --- | --- |
| NM_000238.3 | hKCNH2 | CGTGCTGCCTGAGTACAAGCT | TGAAGACAGCCGTGTAGATGA | 125 |
| NM_004346.3 | hCASP3 | TTTTTCAGAGGGGATCGTTG | cggcctccactggtatttta | 151 |
| NM_001228.4 | hCASP8 | AAGCAAACCTCGGGGATACT | ggggcttgatctcaaaatga | 164 |
| NM_000633.2 | hBcl2 | GAGGATTGTGGCCTTCTTTG | acagttccacaaaggcatcc | 170 |
| NM_138761.3 | hBax | TTTGCTTCAGGGTTTCATCC | atcctctgcagctccatgtt | 162 |
| NM_001101.3 | ACTB | CCTGAACCCTAAGGCCAACCG | GCTCATAGCTCTTCTCCAGGG | 397 |
