# Supplementary material for: Evaluation of the Cardiotoxicity of Mitragynine and Its Analogues Using Human Induced Pluripotent Stem Cell-Derived Cardiomyocytes
Source: PLoS One. 2014 Dec 23;9(12):e115648. doi: 10.1371/journal.pone.0115648 (PMC4275233; doi:10.1371/journal.pone.0115648)
Supplement: S1 Methods — (DOCX) [file pone.0115648.s003.docx]

**METHODS S1**

### Isolation of Mitragynine and its analogues

### The fresh leaves of M. speciosa Korth (Rubiaceae) were collected from the state of Perlis, Malaysia. Authentication of plant material was carried out by a botanist from the Forest Research Institute Malaysia (FRIM). Plant voucher specimen was deposited at the Department of Bio-Screening, IPharm with code number IPHARM-49-35-C1. The fresh leaves were dried using drying oven at 50 °C for a week. Subsequently, the powdered dried material was extracted with methanol for 72 h using soxhlet apparatus. The crude solution was then filtered and evaporated under reduced pressure at 40-45 °C to obtain methanol extract. The extract is dissolved in 10% (v/v) acetic acid and left to stand for 24 h. The solution was then filtered to yield the acidic filtrate, and washed with n-hexane and 25% (v/v) ammonia solution and finally extracted using portions of chloroform. The chloroform extract was mixed with anhydrous sodium sulfate and evaporated to yield crude alkaloid extract. The crude alkaloid was fractionated using SiO_2_ column chromatography with n-hexane and ethyl acetate eluent system. The alkaloids were eluted in order of mitragynine, followed by paynanthiene, speciogynine, and speciociliatine. The eluted fractions were also subjected to preparative- TLC to increase the purity of the compounds. Subsequently, the fractions were evaporated to dryness and recrystallized with n-hexane and diethyl ether to produce an approximately 30% (w/w) of mitragynine, followed by paynanthiene (8% w/w), speciogynine (5% w/w) and speciociliatine (1% w/w). The structure and identity of the compounds confirmed using 1H-NMR and 13C-NMR analysis are shown below.

### Mitragynine

### 1H-NMR (400 MHz, CDCl3 ): δ 7.73 (1H,br-s,NH), 7.43 (1H, s, H-17), 6.99 (1H, t, J=8.0Hz, H-11), 6.90 (1H, d, J=8.0Hz, H-12), 6.45 (1H, d, J=8.0Hz, H-10), 3.87 (3H, s, 9-OCH3), 3.73 (3H, s, 17-OCH3), 3.71 (3H, s, COOCH3), 3.15 (1H,br-dd, J=11.0,2.0Hz,H-3), 3.11 (1H, td, J=12.0, 2.7Hz, 3.03 (1H, dt, J=12.0, 3.6Hz, 2.93-2.99 (3H, m), 2.53 (2H, m), 2.45 (1H,dd,J=11.2, 2.8/2.9 Hz), 1.79 (2H, m), 1.62 (1H, m), 1.20 (1H, m) 0.87 (3H,t,J=7.3Hz, 18-CH3. 13C- NMR (125 MHz, CDCl3 ): δ 169.3 (COOCH3 ), 160.6 (C-17), 154.5 (C-9), 137.2 (C-13), 133.7 (C-2), 121.8 (C-11), 117.7 (C-8), 111.5 (C-16), 107.9 (C-7), 104.2 (C-12), 99.7 (C-10), 61.6 (17-OCH3 ), 61.3 (C-3), 57.8 (C-21), 55.3 (9-OCH3 ), 53.8 (C-5), 51.4 (COOCH3 ), 40.7 (C-20), 39.9 (C-15), 29.9 (C-14), 23.9 (C-6), 19.1 (C-19), 12.9 (C-18).

### Paynanthiene

### 1H-NMR (400 MHz, CDCl3 ): δ 7.70 (1H,br-s,NH), 7.36 (1H, s, H-17), 7.02 (1H, t, J=8.0Hz, H-11), 6.90 (1H, d, J=8.0Hz, H-12), 6.48 (1H, d, J=8.0Hz, H-10), 3.90 (3H, s, 9-OCH3), 3.80 (3H, s, 17-OCH3), 3.71 (3H, s, COOCH3), 3.31 (1H,br-d, J=11.2Hz,H-3), 3.00-3.11 (1H, dd, J=11.3, 3.4 Hz, H-21), 3.08 (1H, m, H-5), 3.00 (1H, m-d, J=15.9 Hz, H-6), 3.03 (1H, m, H-20), 2.55 (1H, ddd, J=11.4, 11.4, 4.4 Hz, H-5), 2.78 (1H, td J=11.6, 3.6Hz, H-15), 2.31 (1H, 2.31, t, J = 12.0 Hz, H-21), 2.11 (1H, m, H2 -14), 5.60(each 1H, m, H2-19), 4.97 (1H,dd,J=10.4Hz, 18-CH3). 13C- NMR (125 MHz, CDCl3 ): δ 169.0 (COOCH3 ), 159.9 (C-17), 154.7 (C-9), 137.2 (C-13), 133.0 (C-2), 122.0 (C-11), 117.7 (C-8), 111.7(C-16), 107.9 (C-7), 104.2 (C-12), 99.8 (C-10), 61.6 (17-OCH3 ), 60.2 (C-3), 61.3 (C-21), 55.4 (9-OCH3 ), 53.2 (C-5), 51.3 (COOCH3 ), 42.8 (C-20), 38.7 (C-15), 33.5 (C-14), 23.7 (C-6), 140.0(C-19), 115.5 (C-18).

### Speciogynine

### 1H-NMR (400 MHz, CDCl3 ): δ 7.75 (1H,br-s,NH), 7.38 (1H, s, H-17), 7.02 (1H, t, J=8.0Hz, H-11), 6.90 (1H, d, J=8.0Hz, H-12), 6.48 (1H, d, J=8.0Hz, H-10), 3.89 (3H, s, 9-OCH3), 3.68 (3H, s, 17-OCH3), 3.68 (3H, s, COOCH3), 3.26 (1H,br-dd, J=11.0Hz, H-3), 3.15-3.26 (1H, dd, J=11.3, 3.4 Hz, H-21), 3.08 (1H, m, H-5), 3.00 (1H, m-d, J=15.9 Hz, H-6), 2.67 (1H, m, H-20), 2.57 (1H, ddd, J=11.4, 11.4, 4.4 Hz, H-5), 2.26 (1H, br, H-15), 2.05 (1H, br-dd, J=11.3, 11.3 Hz, H-21), 2.01 and 1.87 (each 1H, m, H2 -14), 1.06 and 1.44, m (each 1H, m, H2-19), 0.88 (3H,dd,J=7.5Hz, 18-CH3). 13C- NMR (125 MHz, CDCl3 ): δ 171.9 (COOCH3 ), 159.9 (C-17), 154.7 (C-9), 137.5 (C-13), 133.2 (C-2), 122.0 (C-11), 117.7 (C-8), 113.0 (C-16), 108.0 (C-7), 104.2 (C-12), 99.7 (C-10), 61.6 br (17-OCH3 ), 60.4 (C-3), 61.0 (C-21), 55.3 (9-OCH3 ), 53.6 (C-5), 51.6 br (COOCH3 ), 40.3 br (C-20), 38.7 (C-15), 33.8 br (C-14), 23.8 (C-6), 24.5 (C-19), 11.3 (C-18).

### Speciociliatine

### 1H-NMR (400 MHz, CDCl3 ): δ 7.84 (1H,br-s,NH), 7.45 (1H, s, H-17), 7.03 (1H, t, J=8.0Hz, H-11), 6.94 (1H, d, J=8.0Hz, H-12), 6.50 (1H, d, J=8.0Hz, H-10), 3.91 (3H, s, 9-OCH3), 3.80 (3H, s, 17-OCH3), 3.69 (3H, s, COOCH3), 4.20 (1H,br-s, J=11.0 Hz,H-3), 2.89-3.01 (1H, dd, J=11.3, 5.8 Hz, H-21), 3.01 (1H, m, H-5), 3.01 (1H, m-d, J=15.9 Hz, H-6), 1.74(1H, br-s, H-20), 3.14 (1H, dd, J=11.4, 11.4, 4.4 Hz, H-5), 3.01 (1H, br, H-15), 2.80 (1H, br-dd, J=11.3, 11.3 Hz, H-21), 2.64 (1H, m, H2 -14), 1.26 and 1.64, m (each 1H, m, H2-19), 0.90 (3H,dd,J=7.3Hz, 18-CH3). 13C- NMR (125 MHz, CDCl3 ): δ 169.5 (COOCH3 ), 160.1 (C-17), 154.4 (C-9), 137.2 (C-13), 131.8 (C-2), 121.9 (C-11), 117.9 (C-8), 111.3 (C-16), 108.1 (C-7), 104.4 (C-12), 99.7 (C-10), 61.5 (17-OCH3 ), 54.6 (C-3), 52.0 (C-21), 55.3 (9-OCH3 ), 52.4 (C-5), 51.5 (COOCH3 ), 39.6 (C-20), 38.8 (C-15), 29.8 br (C-14), 20.7 br (C-6), 20.6 br (C-19), 12.6 (C-18).

**Cells and cell culture**

### hiPSC-CMs were maintained in the specific medium provided by Cell Dynamic International Inc. Cells were cultured at 37^o^C in a humid CO_2_ incubators supplemented with 5 % CO_2_ and the cultural medium was changed every three days. For Patch-Clamping, hiPSC-CMs were plated into 3.5-cm petri dishes pre-coated with 0.1% (w/v) gelatin. hiPSC-CMs of 5~6 weeks post hiPSCs differentiation were adopted in this study.

### hERG-HEK293 cells were cultured in Dulbecco's modified Eagle medium (DMEM) supplemented with 10% FBS and 1% Penicillin-Streptomycin-Glutamine (all cultural reagents were from Invitrogen, Singapore). Cell viability before and after drug treatments were evaluated using Trypan blue exclusion assay.

**Voltage clamp of hERG-HEK**

hERG current was activated with a step to 20 mV, and tail current was recorded after the step to – 50 mV. The voltage protocol was repeated at 15 seconds intervals (same as for in hiPSC-CM). hERG current was elicited by depolarizing steps to between -50 mV and 50 mV for 4 seconds applied from a holding potential of -80 mV. Tail current was recorded after the repolarizing step to – 50 mV. Cells were superfused with external solution contained (in mM): NaCl 137, KCl 4, CaCl_2_ 1.8, MgCl_2_ 1, HEPES 10, Glucose 10, adjusted to pH 7.40 with NaOH. Pipette solution contained (in mM): potassium aspartate 130, MgCl_2_ 5, EGTA 5, EDTA 0.2, ATP 4, HEPES 10, adjusted to pH 7.20 with KOH.

The steady-state inactivation properties of the *I*_kr_ (hERG) were measured in hERG-HEK293 cells. Normalized steady-state inactivation as a function of pre-pulse of test potential (PT) were fitted to a Boltzmann function. Steady-state inactivation was analyzed as described previously.^1^ Briefly, the corrected steady-state inactivation curves were fitted with a Boltzmann function in the following form: I/(I_max_-I_min_)=I/{1+exp[(V_t_-V_1/2_)/K]}+I_min_. Where I is the amplitude of inactivating current corrected for deactivation, I_max_ is the maximum of I , I_min_ is the minimum of I, V_t_ is the pre-pulse of test potential, V_1/2_ is the voltage at which I is half of max, and k is the slope factor. Experiments were performed at room temperature.

### Reverse Transcriptase Polymerase Chain Reaction (RT-PCR) and real-time PCR

### hiPSC-CMs were exposed to mitragynine at 10 µM for 4 hours. Next, total RNA was extracted by RNeasy Mini kit (QIAGEN, Singapore). First strain cDNA was synthesized by SuperScript^®^ III First-Strand Synthesis System (Invitrogen, Singapore) using oligo dT primer. Human left ventricular tissue mRNA from Clontech (Mountain View, USA) was used as control for RT-PCR. The expression of KCNH2 and apoptotic relate genes (Caspase3, 8, Bax and Bcl2) in hiPSC-CMs were profiled using 2x PCR reaction Mix from Thermo Scientific (Pittsburgh, USA). The PCR of the tested genes was performed for 40 cycles of the following: denaturing at 94^o^C for 30 seconds, annealing at 60^o^C for 30 seconds and extension at 72^o^C for 60 seconds. β-actin (ACTB) PCR was performed for 30 cycles. Quantitative PCR was performed with the ViiA 7 real-time PCR system (Applied Biosystems, Singapore). PCR reactions were prepared with the SYBR® Green PCR Master Mix (Invitrogen, Singapore). Samples were normalized to ACTB and the control value was given as 100%. Samples were plotted relative to that value. The results are representative of three independent experiments. PCR Primers were shown in Table S1.

**Caspase 3 activity assay**

Caspase 3 activity was measured in hiPSC-CMs using a Caspase-3 Fluorescence Assay Kit (Cayman Chemical, Ann Arbor, USA). hiPSC-CMs treated with 10 μM Mitragynine for 0, 4, 8, 12 and 24 hours were lysed and the Caspase 3 activity in the lysate was determined using a fluorescent plate reader.

**Immunocytochemistry**

Disassociated CMs cultured on glass cover slips were exposed to mitragynine for 4 hours then fixed using 4% paraformaldehyde and permeabilized with 1% Triton-X-100. After blocking with 5% goat serum in PBS for 1 h at room temperature, cells were co-stained with antibodies against cardiac sarcomeric alpha-actinin (clone EA-35, Sigma) and KV11.1 (hERG) (extracellular, encoded by KCNH2) (Alomone Labs, Jerusalem, Israel) followed by fluorescent labeled secondary Abs (Alexa 488 and Alexa 555, respectively). Confocal immonofluorescent images were acquired using a LSM-710 laser scanning confocal microscope (Carl Zeiss, Germany).
