# Supplementary material for: Evaluation of the Cardiotoxicity of Mitragynine and Its Analogues Using Human Induced Pluripotent Stem Cell-Derived Cardiomyocytes
Source: PLoS One. 2014 Dec 23;9(12):e115648. doi: 10.1371/journal.pone.0115648 (PMC4275233; doi:10.1371/journal.pone.0115648)
Supplement: S1 Results — (DOCX) [file pone.0115648.s004.docx]

**RESULTS S1**

As we and others have previously reported, ^2-4^ ventricular (V)-like hiPSC-CMs can be easily distinguished from atrial (A)-like and nodal (N)-like hiPSC-CMs by longer APD of 50% and 90% repolarization (APD50 and APD90), which reflects a broader Phase 2 of the AP trace contributed by the L-type Ca^2+^ influx as L-type Ca^2+^ channels that are predominately presented in ventricular cardiomyocytes. On the other hand, AP amplitude (APA) and maximum upstroke velocity (dV/dt_max_) distinguished N-like hiPSC-CMs from V- and A-like hiPSC-CMs. APA in V- and A-like hiPSC-CMs (80~100 mV) is mainly determined by a strong fast inward sodium current during depolarization. In contrast, there is a smaller APA in N-like hiPSC-CMs (~60 mV) due to lack of sodium current in depolarization (Phase 0). Moreover, N-like hiPSC-CMs is characterized with slower dV/dtmax that in corresponding to a slower depolarization process (slower slope of phase 0). Accordingly, we observed that, similar to previous studies, the proportions of V-, A- and N-like hiPSC-CMs observed in hiPSC-CMs were ~75%, ~14% and 11 %, respectively.

**REFERENCES**

1. Smith PL, Baukrowitz T, Yellen G. The inward rectification mechanism of the HERG cardiac potassium channel. Nature. 1996; 379(6568): 833-6.

2. Ma J, Guo L, Fiene SJ, Anson BD, Thomson JA, Kamp TJ, et al. High purity human-induced pluripotent stem cell-derived cardiomyocytes: electrophysiological properties of action potentials and ionic currents. American journal of physiology Heart and circulatory physiology. 2011; 301(5): H2006-17.

3. Hoekstra M, Mummery CL, Wilde AA, Bezzina CR, Verkerk AO. Induced pluripotent stem cell derived cardiomyocytes as models for cardiac arrhythmias. Frontiers in physiology. 2012; 3: 346.

4. Ma D, Wei H, Zhao Y, Lu J, Li G, Sahib NB, et al. Modeling type 3 long QT syndrome with cardiomyocytes derived from patient-specific induced pluripotent stem cells. Int J Cardiol. 2013; 168(6): 5277-86
